# Prioritizing the Lived Experience: The Peer Reviewed Alzheimer’s Research Program

**DOI:** 10.1002/alz.091171

**Published:** 2025-01-09

**Authors:** Sarah N. Fontaine, Katherine Brandt

**Affiliations:** ^1^ Congressionally Directed Medical Research Programs, Fort Detrick, MD USA; ^2^ Massachusetts General Hospital, Boston, MA USA; ^3^ Frontotemporal Disorders Unit, Charlestown, MA USA

## Abstract

The Congressionally Directed Medical Research Programs (CDMRP) originated in 1992 via a Congressional appropriation to foster novel approaches to biomedical research in response to the expressed needs of its stakeholders‐the American public, the military, and Congress. Currently there are 35 CDMRP programs each addressing a specific disease or condition.

The Peer Reviewed Alzheimer’s Research Program (PRARP) began in 2011 and has a vision to mitigate the impact of Alzheimer’s and related dementias associated with military and diverse risks. To do this, the PRARP funds person‐centered research to address critical needs and improve quality of life for people living with Alzheimer’s diseases and related dementias.

A CDMRP hallmark is the inclusion of people with lived experience at all levels. For the PRARP, this means inclusion as an equal voice and vote in setting the annual investment strategy and priority areas for a program, reviewing research applications, and making funding recommendations. They may be a person with a dementia diagnosis, family member, and/or care partner. This collaborative approach brings an important perspective and insight on the needs of the Alzheimer’s and dementia community and brings a sense of urgency to the entire process. The PRARP goes one step further and requires community collaboration in funded research projects. Any project proposing clinical research is required to have community collaboration throughout the design, execution, and dissemination of the research. This talk from the PRARP program manager and a PRARP lived experience peer‐reviewer will describe how people with lived experience are empowered to share their voice and are integrated throughout the PRARP’s processes and funded projects.

